# Development of a high-throughput method to evaluate serum bactericidal activity using bacterial ATP measurement as survival readout

**DOI:** 10.1371/journal.pone.0172163

**Published:** 2017-02-13

**Authors:** Francesca Necchi, Allan Saul, Simona Rondini

**Affiliations:** GSK Vaccines Institute for Global Health (GVGH) S.r.l., Siena, Italy; University Medical Center Utrecht, NETHERLANDS

## Abstract

Serum Bactericidal Activity (SBA) assay is the method of choice to evaluate the complement-mediated functional activity of both infection- and vaccine-induced antibodies. To perform a typical SBA assay, serial dilutions of sera are incubated with target bacterial strains and complement. The conventional SBA assay is based on plating on agar the SBA reaction mix and counting the surviving bacterial colony forming units (CFU) at each serum dilution. Even with automated colony counting, it is labor-intensive, time-consuming and not amenable for large-scale studies. Here, we have developed a luminescence-based SBA (L-SBA) method able to detect surviving bacteria by measuring their ATP. At the end of the SBA reaction, a single commercially available reagent is added to each well of the SBA plate, and the resulting luminescence signal is measured in a microplate reader. The signal obtained is proportional to the ATP present, which is directly proportional to the number of viable bacteria. Bactericidal activity is subsequently calculated. We demonstrated the applicability of L-SBA with multiple bacterial serovars, from 5 species: *Citrobacter freundii*, *Salmonella enterica* serovars Typhimurium and Enteritidis, *Shigella flexneri serovars* 2a and 3a, *Shigella sonnei* and *Neisseria meningitidis*. Serum bactericidal titers obtained by the luminescence readout method strongly correlate with the data obtained by the conventional agar plate-based assay, and the new assay is highly reproducible. L-SBA considerably shortens assay time, facilitates data acquisition and analysis and reduces the operator dependency, avoiding the plating and counting of CFUs. Our results demonstrate that L-SBA is a useful high-throughput bactericidal assay.

## Introduction

The Serum Bactericidal Activity (SBA) assay assesses the complement-dependent bactericidal activity of antibodies in sera against bacterial isolates. This assay is the method of choice to evaluate the complement-mediated functional activity of both infection- and vaccine-induced antibodies [1–3]. For *Neisseria meningitidis*, SBA is the accepted correlate of protection on which meningococcal vaccines are registered [4, 5]. Bactericidal assays have been developed also for other pathogenic microorganisms [6–8] like *Neisseria gonorrhoeae*, *Haemophilus influenza* and *Vibrio cholerae* for which it is considered the gold standard of protection [9]. Additionally, SBA assays have been further used to assess naturally acquired-antibody responses, determining, for example, the relation between age-specific prevalence of specific diseases and presence of bactericidal antibodies [10]. In order to perform a typical SBA assay, serial dilutions of sera are incubated with target bacterial strains and an exogenous source of active complement [4]. Pathogen-specific antibody binds to the bacterial surface via specific protein or carbohydrate moieties. The C1q subunit of C1 complement protein binds to the Fc portion of the surface-bound Ig and such binding activates the classical pathway of complement, which ultimately results in death of the target organism. The serum killing activity is then evaluated by plating the SBA reaction mix and counting the surviving bacterial colony forming units (CFU) at each serum dilution. Bactericidal antibody titers are usually calculated as the reciprocal dilution of test serum that kills ≥ 50% of the bacteria in the assay [11].

The conventional SBA assay is labor-intensive, time-consuming and not amenable to the evaluation of large numbers of serum samples. Although automated colony counting may be used, the process is mostly performed manually, and inter-operator variability is an established source of error when an assay is being qualified for clinical trials. SBA methods have proven difficult to standardize among laboratories [5], and with the method’s cumbersomeness, there is urgent need to develop a sensitive and high-throughput assay for a rapid and robust assessment of presumed vaccine effectiveness. For this reason, recently, fluorescence- or colorimetric-SBA assays have been proposed to replace CFU plate counting [12–14].

In this study, we describe a luminescence-based SBA (L-SBA) assay where bacteria surviving the complement-mediated antibody dependent killing are detected by measuring their metabolic ATP via a novel application of the commercially available BacTiter-Glo Reagent (Promega). The manufacturer describes BacTiter-Glo as a homogenous method for determining the number of viable bacterial cells in culture based on quantitation of the ATP present. This reagent contains a lysis buffer together with a thermostable luciferase and its substrate luciferin that in the presence of ATP is oxidized with the emission of light. L-SBA adheres to the same assay principle as the conventional SBA, but differs in that the bactericidal reaction mix is not plated on agar plates, rather is mixed with this reagent. Bacterial cell lysis is induced and bacterial ATP becomes available to trigger the luciferase-mediated reaction, resulting in a measurable luminescence signal. The signal obtained is proportional to the ATP present in the SBA reaction mix, and is directly proportional to the number of bacteria which were not killed by SBA. Therefore, the bactericidal titer can be calculated directly from the microplate, at the end of the bactericidal reaction, using a luminometer.

We showed that L-SBA can be used with a range of different microorganisms: *Citrobacter freundii*, *Salmonella enterica* serovars Typhimurium and Enteritidis, *Shigella flexneri* serotypes 2a and 3a, *Shigella sonnei*, and *Neisseria meningitidis* serogroups A and W, allowing determination of bactericidal titers of different post-immunization mouse sera. We demonstrated consistent results and a strong correlation between serum titers, as determined by traditional CFU counting method and by L-SBA. We propose L-SBA as a universal alternative readout method for bactericidal reactions against different bacterial species and strains.

## Materials and methods

### Bacterial strains and reagents

All bacterial strains used in this study (described in Table 1) were stored frozen at -80°C in 20% glycerol stocks. *Citrobacter*, *Salmonella* and *Shigella* strains were grown at 37°C in Luria Bertani (LB) medium. In the case of *Shigella* strains, an overnight culture was started from frozen stock cultures. The bacterial suspension was then diluted 1:100 in fresh LB and incubated at 37°C with 180 rpm agitation in an orbital shaker, until they reached an optical density at 600 nm (OD_600_) of 0.2. *Citrobacter* and *Salmonella* strains from frozen glycerol stocks were grown in LB to log phase (0.2 OD_600_) and then diluted to approximately 1 x 10^6^ CFU/mL in phosphate-buffered saline (PBS). Meningococcal strains were grown on GC agar plates (Difco Laboratories, Detroit, Mich.), and the plates were incubated overnight at 37°C in 5% CO_2_. A portion of the plated overnight culture was used to inoculate fresh Mueller Hinton Broth (MHB) (Difco Laboratories, Detroit, Mich.) supplemented with 0.25% (w/v) glucose in a 16 mL-tube. Cultures were incubated at 37°C in 5% CO_2_, shaking, until they reached approximately 0.65 OD_600_. The bacterial suspension was then diluted 1:6 in a new 16 mL-tube with pre-warmed MHB medium supplemented with 0.25% glucose and 0.02 mM Cytidine-5’-monophospho-N-acetylneuraminic acid (CMP-NANA) (Sigma). The cultures were incubated at 37°C in 5% CO_2_ until they reached approximately 0.65 OD_600_. Meningococcal bacteria were then washed once in Dulbecco’s PBS (DPBS) (Sigma) with 1% (w/v) bovine serum albumin (BSA) (Sigma), and then diluted to approximately 1 x 10^6^ CFU/mL in DPBS-1% BSA.

**Table 1:** Bacterial strains used in this study.

Species and serovar	Strain	Isolation location or characteristics	Reference(s) or source
*Citrobacter freundii*	3056	Clinical isolate from Novartis Master Culture Collection	[15]
*Salmonella enterica* serovar Typhimurium	D23580	Clinical isolate from blood culture, Malawi	[10, 16]
*Salmonella enterica* serovar Enteritidis	Ke016	Gastrointestinal isolate, Kenya	[17]
*Shigella flexneri* serotype 2a	142	Clinical isolate	Public Health England (PHE) culture collection
*Shigella flexneri* serotype 3a	144	Clinical isolate	Public Health England (PHE) culture collection
*Shigella sonnei*	71	*S*. *sonnei* 53G Δ*virG*::*cat*	[18]
*Neisseria meningitidis* serogroup A	Niga 16/09	Clinical isolate, Nigeria	Norwegian Institute of Public Health
*Neisseria meningitidis* serogroup W	Mali 4/11	Clinical isolate, Mali	Norwegian Institute of Public Health

### Serum samples

The serum samples used were obtained from mice immunized with conjugates or with Generalized Modules for Membrane Antigens (GMMA)-based vaccines [18, 19]. For tests involving *Citrobacter freundii* 3056 a standard reference pooled serum raised to Vi-CRM_197_ vaccine conjugate [15, 20] was used. Bactericidal activity against *S*. Typhimurium and *S*. Enteritidis was assessed using a standard reference pooled serum raised to *S*. Typhimurium (STm) and *S*. Enteritidis (SEn) GMMA vaccines, respectively. Additionally, individual mouse sera raised to STm and SEn GMMA or O-antigen-CRM_197_ conjugates, with different bactericidal potential, were also included in these tests. Bactericidal activity against *Shigella flexneri* and *Shigella sonnei* [18] was assessed using three reference standard pooled sera raised against GMMA purified from the homologous strains. Bactericidal activity against *Neisseria meningitidis* was assessed using mouse pooled sera raised to different doses of meningococcal GMMA. Additionally, anti-capsular monoclonal antibodies (mAb), specific for serogroups A and W (generously provided by Dan Granoff and Jo Anne Welsch, CHORI, US), were also used. All sera tested in SBA were previously heat-inactivated (HI) at 56°C for 30 minutes to remove endogenous complement activity.

### Conventional SBA

Log-phase cultures were prepared as described above and diluted to approximately 1 x 10^6^ CFU/mL in the assay diluent (PBS or DPBS-1% BSA for *N*. *menigitidis*). The reaction mixtures were prepared in a 96-well round bottom microtiter plate in a final volume of 100 μL, containing 10 μL of the target bacterial cells, 10 μL of HI serum or mAb (2-fold or 1.5-fold serially diluted) and baby rabbit serum (BRS) as external source of complement (Pelfreeze from Invitrogen and Cederlane from Euroclone). For each tested strain we initially determined the optimal lot and percentage of BRS to be used, which lacked intrinsic bactericidal activity. Specifically, we used 5% BRS for *C*. *freundii*, 15% BRS for *S*. *flexneri*, 20% BRS for *S*. *sonnei* and *N*. *menigitidis* strains, and finally 50% BRS for *S*. Typhimurium and Enteritidis strains, as previously published [21]. Negative controls were also included, represented by active BRS without any antibody source and HI BRS. The plates containing the reaction mixtures were incubated for 3 hours at 37°C, in air with 5% CO_2_ for *N*. *meningitidis* or without added CO_2_ for other bacteria. At time zero (T0) and at the end of incubation, serial dilutions of the reactions were spread onto LB or GC-agar plates and grown overnight. Colonies were counted after 16 h incubation and SBA titers were calculated as the reciprocal serum dilution necessary to obtain 50% reduction in relation to the maximum growth of bacteria obtained in the test [1, 22].

### Luminescent-SBA (L-SBA)

L-SBA adheres to the same assay principles as the conventional SBA, but diverges in the readout method. The reaction mixtures were prepared in a 96-well microtiter plate as described above. At the end of the bactericidal reaction, the SBA plate was centrifuged at room temperature (RT) for 10 minutes at 3220×*g*. The supernatant was discarded from all the wells to remove ATP derived from dead bacteria and SBA reagents, and the remaining live bacterial pellets were resuspended in PBS or DPBS-1% BSA in the case of *N*. *meningitidis*. From each well, 50 μL of bacterial suspension was transferred to a white flat-bottom 96-well microtiter plate (Greiner) and 50 μL of the commercially available BacTiter-Glo Reagent (Promega) was added to each well. The reaction was incubated for 5 minutes at RT on an orbital shaker, and then the luminescence signal measured by a luminometer (Victor 2, Wallac from PerkinElmer). In the same way, luminescence was acquired prior to start incubation, at time zero (T0), in a well containing bacteria and buffer, in order to estimate the signal released from the initial concentration of bacteria. The luminometer expresses luminescence as counts per second (CPS). For each well, the average CPS of three consecutive measurements per well was used, using the software Wallac 1420 Workstation. A blank well containing only dilution buffer was included in each plate and the background CPS was subtracted from the samples CPS values. For each tested serum, a bacterial growth inhibition curve was obtained plotting luminescence signal at each serum dilution. As for the conventional SBA assay, bactericidal serum titers were calculated as the reciprocal serum dilution necessary to obtain 50% bacterial growth inhibition.

### Curve fitting and statistical analysis

Raw values from each bactericidal reaction in the presence of test serum (CPS_test180_) were normalized to the maximum bacterial growth obtained after 3 h (180 min, T180) incubation (CPS_max180_), by calculating CPS_test180_/CPS_max180_ or CFU_test180_/CFU_max180_. Normalized data were then analyzed by a four-parameter logistic (4-PL) curve fitting model, using GraphPad Prism 6 software (GraphPad Software, La Jolla, CA). The reciprocal serum dilution that resulted in a 50% reduction of luminescence or CFU counts normalized per the maximum signal (in terms of luminescence or CFU counts detected at the highest serum dilution, equal to the signal given by the complement controls) was reported as the titer of the serum sample, as well as for mAb when used in the experiments. The normalization was convenient to plot multiple data sets on the same scale and had no impact in determining the bactericidal titer. However, we noticed that the variation in the maximum growth of the different bacteria in the assay condition influences the baseline of the bactericidal curves, which may vary between 0.1 (less bacterial growth) and 0.01 (higher bacterial growth).

To evaluate reproducibility of SBA assays, 3 independent experiments were performed and mean titers value ± standard deviation (SD) was determined for each serum from the three experiments. Inter-assay variation was also calculated and reported as coefficient of variation (CV) which is the standard deviation divided by the mean. Serum titers from triplicates were assessed and reported with the 95% Confidence Interval (CI) limit values.

To examine the correlation between serum titers determined by traditional CFU counting method and by L-SBA, the results were plotted against each other, and Pearson correlation coefficient (*r*) and *P* value were obtained using GraphPad Prism 6 software.

### Ethical statement

All animal sera used in this study derived from mouse immunization experiments performed at the Novartis Vaccines Animal Facility in Siena, Italy, (now acquired by the GSK group) in compliance with the relevant guidelines of Italy (Italian Legislative Decree n. 116/1992) and the institutional policies of Novartis Vaccines (now acquired by the GSK group). The animal protocol was approved by the Animal Welfare Body of Novartis Vaccines, Siena, Italy, (now acquired by the GSK group) and by the Italian Ministry of Health (Approval number AEC201018).

## Results

### Determination and optimization of assay parameter values

#### Bacterial cell concentration, sensitivity and linearity range

**Fig 1:**
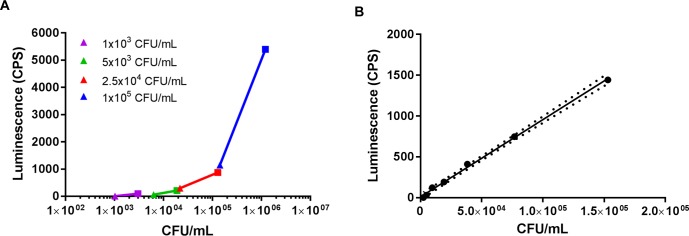
Optimization of bacterial cell concentration for L-SBA. (A) Different bacterial concentrations were tested with 5% BRS and measured by luminescence at T0 (triangle symbol) and at T180 (square symbol). (B) Bacterial cells at 1.5 x 10^5^ CFU/mL were diluted down until 1.0 x 10^3^ CFU/mL and measured by luminescence. The area within the dashed lines define the confidence interval of the best-fit line of the linear regression (y = 0.009465x + 10.28). CPS, counts per second.

The first step to develop a useful readout method, to allow bacteria quantification directly from the wells where the SBA reaction has taken place, consisted of determining the optimal bacterial cell concentration to maximize reagent’s sensitivity. For these preliminary set up tests, we decided to use *Citrobacter freundii*, being an easy to handle BSL-1 microorganism. With the aim of assessing the viability and growth of bacteria, different bacterial concentrations, ranging from 1 x 10^3^ to 1 x 10^5^ CFU/mL were tested in a SBA-like reaction mixture, containing 5% BRS as complement source (and growth factor) in the assay diluent only, without serum. Luminescence was measured following the addition of BacTiter-Glo Reagent at T0 and at T180. Bacterial CFU were also counted on agar-plates the following day. As shown in Fig 1A, the highest span of luminescence between T0 and T180 was obtained using 1 x 10^5^ CFU/mL bacteria in the well (4252 CPS difference compared to 91 CPS difference when 1 x 10^3^ CFU/mL were used at T0, see S1 Table). When bacterial cells were diluted from 1.5 x 10^5^ CFU/mL down to 1 x 10^3^ CFU/mL, a very good linear relationship between luminescence and cell concentrations by CFU counts was found (*r* > 0.99, *p* < 0.0001) (Fig 1B), hence 1 x 10^5^ CFU/mL was selected as suitable bacterial concentration for L-SBA setup. Higher bacterial concentrations were not tested because we have already shown that higher starting CFU/mL translates into a higher resistance to complement-mediated killing, thus requiring very high amount of BRS (75%) [23].

#### L-SBA initial set up using *C*. *freundii*: Circumventing serum interference with luminescence readout

**Fig 2:**
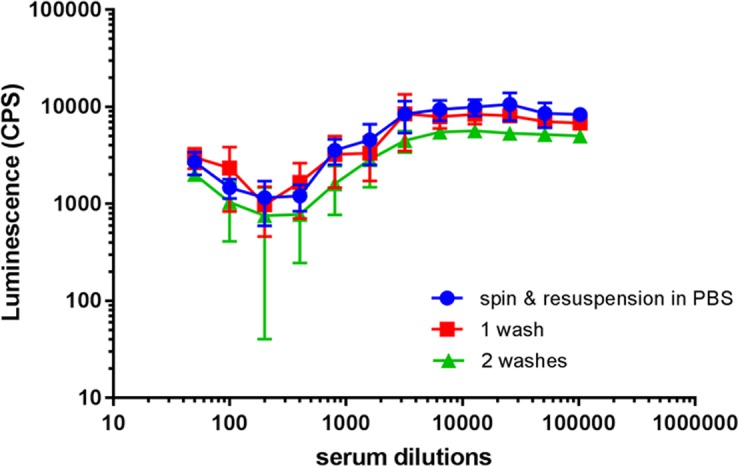
L-SBA optimization to circumvent serum interference with luminescence readout. Bactericidal reaction was tested using *C*. *freundii* in the presence of 5% BRS and serial dilutions of HI standard serum. At the end of the SBA reaction, we performed: a. one spin and resuspension of bacterial pellets in PBS; b. one wash; c. two washes in PBS, before measuring luminescence. Average luminescence signals and standard deviations are shown from three independent experiments, for each condition (for some points the error bars are not visible because shorter than the symbols).

In the initial setup tests, we observed a high interference in the luminescence acquisition caused by the reagents used in the SBA reaction mixture, especially by the BRS when used at high concentrations (50%) (S1 Fig). More specifically, we found that measuring by luminescence the killing effect of 50% BRS for *Citrobacter freundii* (BRS at this concentration is toxic for the microorganism, inducing 99% killing), luminescence was overestimated and unable to reliably quantify the surviving bacteria, while, adding one single washing step, it decreased by almost three times (252 CPS instead of 796 CPS), thus correlating with the expected CFU (S1 Fig). Several tests were performed with *C*. *freundii* in the presence of 5% BRS and serial dilutions of HI standard reference serum (Fig 2) to determine the optimum washing procedure for the elimination of the interference and the reproducibility of measuring the collected live bacteria. Each condition was repeated in three independent experiments. We found that a single spin and resuspension in PBS was sufficient to remove background interference. By contrast, adding more washes with PBS led to loss of bacterial cells and higher standard deviation bars, hence an increased variability in the bactericidal curves (Fig 2). In the bactericidal curves of *C*. *freundii* (Fig 2) a prozone effect was observed at higher serum concentrations (50- and 100-fold dilutions), where the signal was higher compared to what found at lower concentration (200-fold dilution). Such phenomenon may be due to either antibody excess or presence of nonspecific inhibitors in serum, which may inhibit the killing exerted at high serum concentrations [24].

### Comparison of assays

The relative performance of L-SBA including a single spin and resuspension was assessed for reproducibility, robustness, and comparison with the results of the conventional SBA. SBA assays were tested for clinically relevant pathogenic strains, such as *S*. Typhimurium, *S*. Enteritidis, *Shigella flexneri* serotypes 2a and 3a, *Shigella sonnei* and *Neisseria meningitidis* serogroups A and W.

#### L-SBA evaluation using *Shigella flexneri* 2a and 3a and standard reference sera

**Fig 3:**
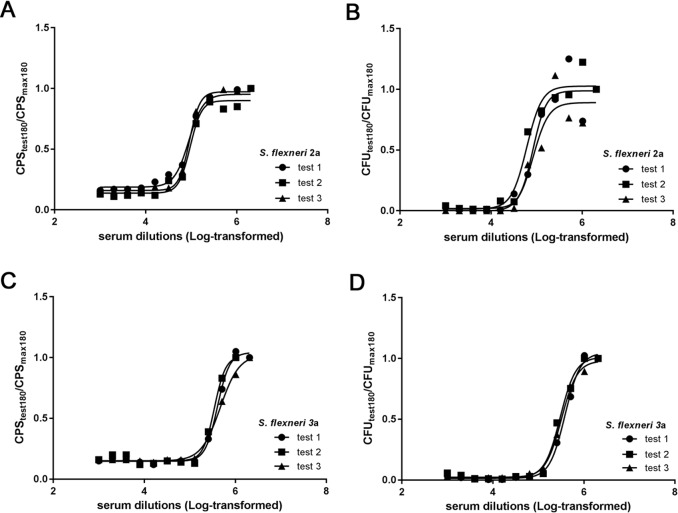
Comparison of L-SBA and conventional SBA assay with standard reference sera against *Shigella flexneri* serogroups 2a and 3a. HI standard reference serum against *S*. *flexneri* 2a (A and B) and *S*. *flexneri* 3a (C and D) was tested by L-SBA (A and C, respectively) and by the conventional assay (B and D, respectively) in three independent experiments. Black lines represent the curve fitting by nonlinear regression for each replicate.

Reference standard sera raised to the homologous strains (strain 142 for *S*. *flexneri* 2a and strain 144 for *S*. *flexneri* 3a) were tested, with each bactericidal reaction simultaneously assayed by both the conventional SBA (plating and CFU counts), and L-SBA. Similar bactericidal curves were obtained when SBAs against *S*. *flexneri* 2a or 3a were analyzed in terms of CFU counts and of luminescence signal (Fig 3), and good fits to a 4 parameter curve were obtained, especially using luminescence signal (*R*^*2*^ > 0.98) (Table 2). Similar serum titers were obtained by both methods, as shown in Table 2. L-SBA was able to generate reproducible results, with very low variability especially with *S*. *flexneri* 2a (3% coefficient of variation CV compared to 17% CV for CFU titer) (Table 2).

**Table 2:** SBA titers against *S*. *flexneri* 2a and 3a as determined by L-SBA and by CFU counts-based method.

	test 1	test 2	test 3	Average	SD	CV %
***S*. *flex 2a***						
**L-SBA titer**	88662	94734	91261	**91552**	**3046**	**3**
*R*^*2*^	*0*.*9819*	*0*.*9820*	*0*.*9957*			
**CFU titer**	82978	60520	83720	**75739**	**13186**	**17**
*R*^*2*^	*0*.*9423*	*0*.*9703*	*0*.*9230*			
***S*. *flex 3a***				** **	** **	** **
**L-SBA titer**	403554	345048	464769	**404457**	**59866**	**15**
*R*^*2*^	*0*.*9954*	*0*.*9906*	*0*.*9974*			
**CFU titer**	382494	301295	320411	**334733**	**42452**	**13**
*R*^*2*^	*0*.*9965*	*0*.*9923*	*0*.*9980*			

#### Bactericidal titers from L-SBA correlate with titers obtained in conventional SBA, using anti-STm and anti-SEn individual sera

**Fig 4:**
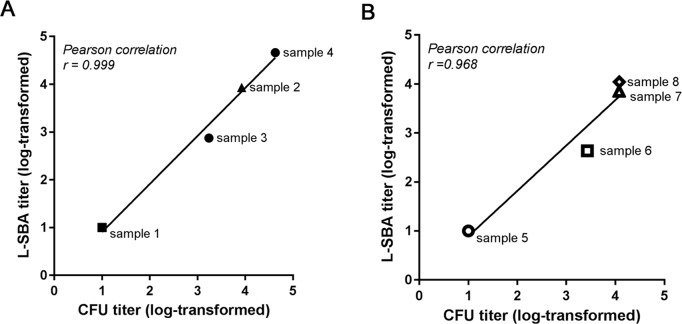
**Correlation of serum titers from individual mouse sera against *S*. Typhimurium (A) and *S*. Enteritidis (B) as determined by L-SBA and by the conventional SBA assay.** Individual mouse sera were simultaneously assayed by L-SBA (y-axis) and by the conventional CFU-based assay (x-axis) against *S*. Typhimurium (A) and against *S*. Enteritidis (B). Plots compare titers from each assay and demonstrate a linear correlation with a slope of 1. Black lines represent the fitting by linear regression. SBA titers were obtained by single experiments.

To determine the relationship between the serum titers obtained in L-SBA and the titers from a conventional assay, a panel of eight individual mouse sera (4 raised against STm and other 4 raised against SEn) covering a broad range of titers was simultaneously assayed with the two methods (Fig 4). Plotting in the same graph the CFU counts based titers (*x* axis) and the L-SBA titers (*y* axis), we obtained good agreement between the two assays, as demonstrated by the Pearson correlation coefficient: *r =* 0.999, *p* < 0.001 for STm SBA; *r* = 0.968, *p* < 0.05 for SEn SBA (Fig 4 and Table 3), indicating a linear correlation.

**Table 3:** Linear regression values for data shown in Fig 4.

Serovar		*y*^*1*^	
	*a*	*b*	*R*^*2*^
STm	1.006	-0.098	0.986
SEn	0.927	-0.034	0.938

^1^ y = ax + b, where a is the slope and b is the y intercept

#### L-SBA can be applied to multiple bacterial species

**Fig 5:**
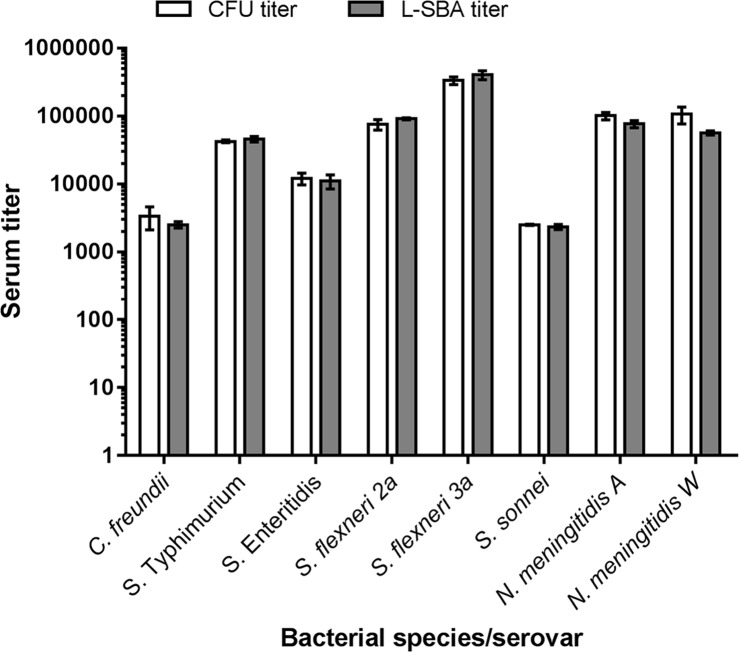
Comparison of titers obtained by the conventional SBA assay and by L-SBA, using a panel of standard reference sera or mAbs against different bacterial species. White or grey bars represent the SBA titers calculated from the conventional CFU-counts or from L-SBA, respectively. Standard reference sera were used against *C*. *freundii*, *S*. Typhimurium, *S*. Enteritidis, *S*. *flexneri 2a and 3a* and *S*. *sonnei*. For *N*. *meningitidis* A and W anti-capsular mAbs were used. The results are the mean ± standard deviation values from three independent experiments.

To demonstrate that L-SBA can generate reproducible data, applicable to different strains and bacteria, we enlarged the panel of bacterial species tested by L-SBA. In addition to SBA against *C*. *freundii* 3056, *S*. *flexneri* 2a 142, *S*. *flexneri* 3a 144, STm D23580 and SEn Ke016, we setup bactericidal assays against *S*. *sonnei* (using the in-house made strain 71, where *virG* gene was substituted with the chloramphenicol resistance cassette to stabilize O-antigen expression) [18], and against *Neisseria meningitidis* serogroups A and W, using the invasive African strains Niga 16/09 and Mali 4/11, respectively. A standard reference pooled serum was used for the SBA assay against *S*. *sonnei*. For *N*. *meningitidis* seroroup A and W, we used mAbs against the capsular polysaccharide types A and W, respectively. Fig 5 shows the complete panel of the bacterial species tested in our study using standard reference sera or mAbs as a source of bactericidal antibodies. The serum titers obtained by L-SBA were similar (almost identical for *S*. Typhimurium, *S*. Enteritidis and *S*. *sonnei)* to the titers obtained by the conventional CFU-based assay (Fig 5 and Table 4). Most importantly, we observed a very low variability among the replicates of L-SBA titers (Fig 5 and Table 4).

**Table 4:** Serum titers and standard deviation (SD) values shown in Fig 5.

	*C*. *freundii*			*S*. Typhimurium			*S*. Enteritidis			*S*. *flexneri 2a*			*S*. *flexneri 3a*			*S*. *sonnei*			*N*. *meningitidis A*			*N*. *meningitidis W*		
	Mean	SD	CV %	Mean	SD	CV %	Mean	SD	CV %	Mean	SD	CV %	Mean	SD	CV %	Mean	SD	CV %	Mean	SD	CV %	Mean	SD	CV %
**CFU titer**	3367	1260	37	42438	1984	5	12073	2385	20	75739	13186	17	334733	42452	13	2505	54	2	101058	12723	13	106520	29899	28
**L-SBA titer**	2517	277	11	45917	4335	9	11022	2579	23	91552	3046	3	404457	59866	15	2331	206	9	76564	9390	12	56651	3981	7

#### Assessment of sera with different bactericidal potential against *N*. *meningitidis* by L-SBA

**Fig 6:**
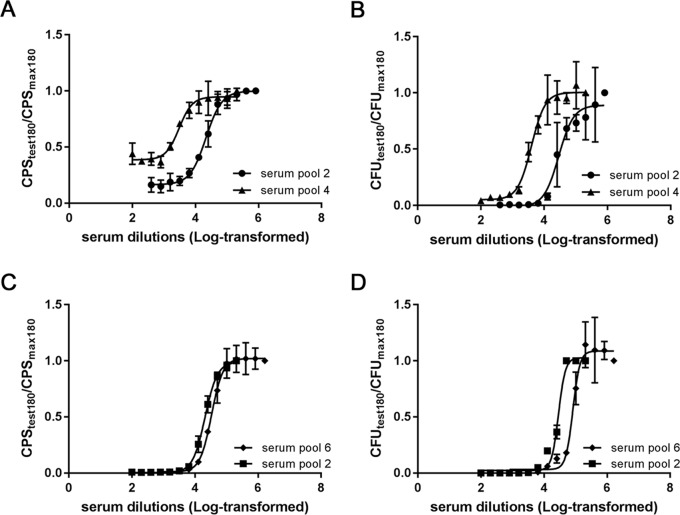
Comparison of L-SBA and conventional assay using pooled mouse sera against *Neisseria meningitidis* serogroups A and W. HI pooled mouse sera were tested by L-SBA (A, C) or by conventional assay (B, D) against *N*. *meningitidis* A Niga 16/09 strain (A, B) or *N*. *meningitidis* W Mali 4/11 strain (C, D). Results are the mean ± standard deviation values from three replicates (for some points the error bars are not visible because shorter than the symbols).

With *N*. *meningitidis* A and W strains, two additional pooled mouse sera, raised to meningococcal GMMA were also assayed. For the SBA with *N*. *meningitidis* A, serum pool n° 4 was raised to a lower dose of GMMA (0.2 μg), consequently, as expected, showed less bactericidal activity than serum pool n° 2, raised to a higher dose (5 μg). The two SBA methods gave similar bactericidal curves for the serum pool 4 and similar curves for the serum pool 2, with a lower variability among the replicates of L-SBA (Fig 6A and 6B and Table 5). Similarly for *N*. *meningitidis* W, we selected two pooled mouse sera raised to different meningococcal GMMA with different bactericidal activity and obtained similar results in the comparison to that obtained with the anti-*N*. *meningitidis* A sera pools (Fig 6C and 6D).

**Table 5:** Serum titers with 95% Confidence Interval (CI) limits from bactericidal reactions shown in Fig 6 against *N*. *meningitidis* A (MenA) and *N*. *meningitidis* W (MenW).

**MenA**	**serum pool 2**			**serum pool 4**		
	**Mean**	**95% CI Upper limit**	**95% CI Lower limit**	**Mean**	**95% CI Upper limit**	**95% CI Lower limit**
**L-SBA titer**	21661	25438	18415	3044	4121	2345
**CFU titer**	28892	48280	20477	3943	4928	3175
**MenW**	**serum pool 6**			**serum pool 2**		
	**Mean**	**95% CI Upper limit**	**95% CI Lower limit**	**Mean**	**95% CI Upper limit**	**95% CI Lower limit**
**L-SBA titer**	33481	38057	29447	20881	22354	19544
**CFU titer**	82178	nd	69976	28803	30942	26605

## Discussion

SBA assay is the method of choice to evaluate the complement-mediated functional activity of vaccine-induced antibodies targeting several pathogenic microorganisms [1, 25, 26], and for organisms such as *N*. *meningitidis* serogroup B, it is considered the gold standard correlate of protection [5].

In the present study, we successfully tested L-SBA with multiple Gram-negative bacteria: *Citrobacter freundii*, *Salmonella* Typhimurium and Enteritidis, *Shigella flexneri* serogroups 2a and 3a, *Shigella sonnei*, and *Neisseria meningitidis* serogroups A and W, demonstrating a broad applicability of using an ATP detection method for quantifying surviving bacteria. Most important with all the target bacteria tested, we obtained highly reproducible data and a strong correlation between serum titers, as determined by traditional CFU counting method and by L-SBA.

Because the L-SBA eliminates the labor-intensive steps of plating the reaction mixtures and counting individual CFU, it brings significant advantages over the conventional assay (Table 6) in terms of: 1. reduced *in vitro* assay time; 2. faster data acquisition and analysis, as the luminescence signal in the SBA reaction is directly measured by a microplate reader instead of CFU counting; 3. high reproducibility and operator independency, as bacteria quantitation does not rely upon plating of serial dilutions of the reaction mixtures; 4. higher throughput: in our standard manual L-SBA we routinely test 24 serially diluted individual sera at 8 different concentrations in triplicate (576 data points, not counting controls) in one day. By contrast, in the conventional SBA assay the number of sera to be tested in a manual assay is limited by the number of agar plates to be plated and counted, and in our standard conventional SBA we are able to test 12 sera with 8 dilutions each with a single replicate (96 data points not counting controls) over two days. Furthermore, unlike the conventional SBA that even with automation suffers of major limits in handling large number of plates, the L-SBA assay may further increase its throughput through the use of robotic systems.

**Table 6:** Comparison between L-SBA and traditional CFU-based assay in terms of procedure advantages.

	L-SBA	traditional SBA with manual counts
**Total time of execution**	6 hours^1^	1.5 working day^2^
**Data acquisition**	2 minutes/SBA plate	2–3 hours/SBA plate^2^
**Reproducibility**	higher operator independency	lower operator independency
**Assay throughput**	1 operator/day: 24 individual sera in triplicate (6 SBA plates total)	1 operator/1.5 day: 12 individual sera in single (1 SBA plate^2^)

^1^to execute 1 set of 6 SBA plates

^2^to execute 1 SBA plate, plating each reaction well in 1 full agar plate: 1 SBA plate corresponds to have 96 agar plates

Previous attempts to develop non-agar-based readout methods for bactericidal reactions were based on fluorescence or colorimetry, e.g., the metabolic indicator alamarBlue [14, 27], that in the presence of cellular respiration is reduced, exhibiting both a fluorescence change and a colorimetric change. However, those methods require further incubation or kinetic measurement of the bacteria at the end of the bactericidal reactions [27] and cannot be performed without automatized robotic equipment. On the contrary, L-SBA is an end-point assay and results are acquired and analyzed on the same day of the assay execution, with no dependence on special equipment but a luminometer microplate reader.

Collectively, our results demonstrate that L-SBA is a sensitive, reproducible and easy to perform method, particularly promising for large-scale studies, which are unfeasible using the traditional agar plate-based assay. Possible applications may include: testing panels of bacterial strains, representatives of different antigens structure and fine antibody specificities; screening high number of human sera as possible complement sources; evaluating correlates of protection in animal models or monitor how SBA specificities change along with antibody maturation and immunoglobulin subclasses; conducting epidemiological studies with large panels of isolates to screen their antigenic diversity and other factors such as sensitivity to SBA. Ultimately, in late stage vaccine development, a rapid and robust SBA method may guide identification of best formulation and optimal dose and schedule, and more importantly may be part of large vaccine efficacy trials.

## Supporting information

S1 Table Raw values and calculated span in luminescence and CFU/mL for data shown in Fig 1A. (PDF)

S1 Fig Interference of BRS with luminescence reading. Without washing steps, increasing concentrations of BRS led to high luminescence values (up to 978 CPS using 50% BRS), compared to 5% BRS (252 CPS) (circle ●). Measuring by luminescence the killing effect of 50% BRS for *C*. *freundii* (inducing 99% killing), we observed that, without washing step, luminescence was overestimated (square ■), while one washing step decreased the value by almost three times (triangle ▲).
(TIF)

## References

[1] Boyd MA, Tennant SM, Saague VA, Simon R, Muhsen K, Ramachandran G, et al Serum bactericidal assays to evaluate typhoidal and nontyphoidal Salmonella vaccines. Clin Vaccine Immunol. 2014;21(5):712–21. PubMed Central PMCID: PMCPMC4018884. 10.1128/CVI.00115-14 24623629 PMC4018884

[2] Campbell H, Borrow R, Salisbury D, Miller E. Meningococcal C conjugate vaccine: the experience in England and Wales. Vaccine. 2009;27 Suppl 2:B20–9. 19477053 10.1016/j.vaccine.2009.04.067

[3] Gill CJ, Baxter R, Anemona A, Ciavarro G, Dull P. Persistence of immune responses after a single dose of Novartis meningococcal serogroup A, C, W-135 and Y CRM-197 conjugate vaccine (Menveo(R)) or Menactra(R) among healthy adolescents. Hum Vaccin. 2010;6(11):881–7. PubMed Central PMCID: PMCPMC3060384. 10.4161/hv.6.11.12849 21339701 PMC3060384

[4] Goldschneider I, Gotschlich EC, Artenstein MS. Human immunity to the meningococcus. I. The role of humoral antibodies. J Exp Med. 1969;129(6):1307–26. PubMed Central PMCID: PMCPMC2138650. 4977280 10.1084/jem.129.6.1307 PMC2138650

[5] Borrow R, Carlone GM, Rosenstein N, Blake M, Feavers I, Martin D, et al Neisseria meningitidis group B correlates of protection and assay standardization—international meeting report Emory University, Atlanta, Georgia, United States, 16–17 March 2005. Vaccine. 2006;24(24):5093–107. 16838413 10.1016/j.vaccine.2006.03.091

[6] Elkins C, Carbonetti NH, Varela VA, Stirewalt D, Klapper DG, Sparling PF. Antibodies to N-terminal peptides of gonococcal porin are bactericidal when gonococcal lipopolysaccharide is not sialylated. Mol Microbiol. 1992;6(18):2617–28. 1280317 10.1111/j.1365-2958.1992.tb01439.x

[7] Green BA, Quinn-Dey T, Zlotnick GW. Biologic activities of antibody to a peptidoglycan-associated lipoprotein of Haemophilus influenzae against multiple clinical isolates of H. influenzae type b. Infect Immun. 1987;55(12):2878–83. PubMed Central PMCID: PMCPMC260001. 3316025 10.1128/iai.55.12.2878-2883.1987 PMC260001

[8] Mountzouros KT, Kimura A, Cowell JL. A bactericidal monoclonal antibody specific for the lipooligosaccharide of Bordetella pertussis reduces colonization of the respiratory tract of mice after aerosol infection with B. pertussis. Infect Immun. 1992;60(12):5316–8. PubMed Central PMCID: PMCPMC258314. 1452367 10.1128/iai.60.12.5316-5318.1992 PMC258314

[9] Son MS, Taylor RK. Vibriocidal assays to determine the antibody titer of patient sera samples. Curr Protoc Microbiol. 2011;Chapter 6:Unit6A 3. PubMed Central PMCID: PMCPMC3228410. 10.1002/9780471729259.mc06a03s23 PMC3228410 22045586

[10] MacLennan CA, Gondwe EN, Msefula CL, Kingsley RA, Thomson NR, White SA, et al The neglected role of antibody in protection against bacteremia caused by nontyphoidal strains of Salmonella in African children. J Clin Invest. 2008;118(4):1553–62. PubMed Central PMCID: PMCPMC2268878. 10.1172/JCI33998 18357343 PMC2268878

[11] McIntosh ED, Broker M, Wassil J, Welsch JA, Borrow R. Serum bactericidal antibody assays—The role of complement in infection and immunity. Vaccine. 2015;33(36):4414–21. 10.1016/j.vaccine.2015.07.019 26187262

[12] Mountzouros KT, Howell AP. Detection of complement-mediated antibody-dependent bactericidal activity in a fluorescence-based serum bactericidal assay for group B Neisseria meningitidis. J Clin Microbiol. 2000;38(8):2878–84. PubMed Central PMCID: PMCPMC87135. 10921943 10.1128/jcm.38.8.2878-2884.2000 PMC87135

[13] Rodriguez T, Lastre M, Cedre B, Fajardo EM, del Campo J, Delgado I, et al Validation of colorimetric assay to detect complement-mediated antibody-dependent bactericidal activity against serogroups B and C Neisseria meningitidis. Biologicals. 2003;31(3):209–12. 12935810 10.1016/s1045-1056(03)00060-5

[14] Romero-Steiner S, Spear W, Brown N, Holder P, Hennessy T, Gomez De Leon P, et al Measurement of serum bactericidal activity specific for Haemophilus influenzae type b by using a chromogenic and fluorescent metabolic indicator. Clin Diagn Lab Immunol. 2004;11(1):89–93. PubMed Central PMCID: PMCPMC321360. 10.1128/CDLI.11.1.89-93.2004 14715550 PMC321360

[15] Rondini S, Micoli F, Lanzilao L, Pisoni I, Di Cioccio V, Saul AJ, et al Characterization of Citrobacter sp. line 328 as a source of Vi for a Vi-CRM(197) glycoconjugate vaccine against Salmonella Typhi. J Infect Dev Ctries. 2012;6(11):763–73. 10.3855/jidc.2495 23277501

[16] Kingsley RA, Msefula CL, Thomson NR, Kariuki S, Holt KE, Gordon MA, et al Epidemic multiple drug resistant Salmonella Typhimurium causing invasive disease in sub-Saharan Africa have a distinct genotype. Genome Res. 2009;19(12):2279–87. PubMed Central PMCID: PMCPMC2792184. 10.1101/gr.091017.109 19901036 PMC2792184

[17] Onsare RS, Micoli F, Lanzilao L, Alfini R, Okoro CK, Muigai AW, et al Relationship between antibody susceptibility and lipopolysaccharide O-antigen characteristics of invasive and gastrointestinal nontyphoidal Salmonellae isolates from Kenya. PLoS Negl Trop Dis. 2015;9(3):e0003573 PubMed Central PMCID: PMCPMC4352093. 10.1371/journal.pntd.0003573 25739091 PMC4352093

[18] Gerke C, Colucci AM, Giannelli C, Sanzone S, Vitali CG, Sollai L, et al Production of a Shigella sonnei Vaccine Based on Generalized Modules for Membrane Antigens (GMMA), 1790GAHB. PLoS One. 2015;10(8):e0134478 PubMed Central PMCID: PMCPMC4527750. 10.1371/journal.pone.0134478 26248044 PMC4527750

[19] Berlanda Scorza F, Colucci AM, Maggiore L, Sanzone S, Rossi O, Ferlenghi I, et al High yield production process for Shigella outer membrane particles. PLoS One. 2012;7(6):e35616 PubMed Central PMCID: PMCPMC3368891. 10.1371/journal.pone.0035616 22701551 PMC3368891

[20] Rondini S, Micoli F, Lanzilao L, Hale C, Saul AJ, Martin LB. Evaluation of the immunogenicity and biological activity of the Citrobacter freundii Vi-CRM197 conjugate as a vaccine for Salmonella enterica serovar Typhi. Clin Vaccine Immunol. 2011;18(3):460–8. PubMed Central PMCID: PMCPMC3067394. 10.1128/CVI.00387-10 21248155 PMC3067394

[21] Rondini S, Lanzilao L, Necchi F, O'Shaughnessy CM, Micoli F, Saul A, et al Invasive African Salmonella Typhimurium induces bactericidal antibodies against O-antigens. Microb Pathog. 2013;63:19–23. 10.1016/j.micpath.2013.05.014 23756206

[22] Jang MS, Sahastrabuddhe S, Yun CH, Han SH, Yang JS. Serum bactericidal assay for the evaluation of typhoid vaccine using a semi-automated colony-counting method. Microb Pathog. 2016;97:19–26. PubMed Central PMCID: PMCPMC4944902. 10.1016/j.micpath.2016.05.013 27216239 PMC4944902

[23] Goh YS, MacLennan CA. Invasive African nontyphoidal Salmonella requires high levels of complement for cell-free antibody-dependent killing. J Immunol Methods. 2013;387(1–2):121–9. 10.1016/j.jim.2012.10.005 23085530

[24] Jacobs JF, van der Molen RG, Bossuyt X, Damoiseaux J. Antigen excess in modern immunoassays: to anticipate on the unexpected. Autoimmun Rev. 2015;14(2):160–7. 10.1016/j.autrev.2014.10.018 25461469

[25] Yang JS, Kim HJ, Yun CH, Kang SS, Im J, Kim HS, et al A semi-automated vibriocidal assay for improved measurement of cholera vaccine-induced immune responses. J Microbiol Methods. 2007;71(2):141–6. 10.1016/j.mimet.2007.08.009 17888533

[26] Maslanka SE, Gheesling LL, Libutti DE, Donaldson KB, Harakeh HS, Dykes JK, et al Standardization and a multilaboratory comparison of Neisseria meningitidis serogroup A and C serum bactericidal assays. The Multilaboratory Study Group. Clin Diagn Lab Immunol. 1997;4(2):156–67. PubMed Central PMCID: PMCPMC170495. 9067649 10.1128/cdli.4.2.156-167.1997 PMC170495

[27] Mak PA, Santos GF, Masterman KA, Janes J, Wacknov B, Vienken K, et al Development of an automated, high-throughput bactericidal assay that measures cellular respiration as a survival readout for Neisseria meningitidis. Clin Vaccine Immunol. 2011;18(8):1252–60. PubMed Central PMCID: PMCPMC3147359. 10.1128/CVI.05028-11 21715580 PMC3147359

