# Supplementary material for: Development of a high-throughput method to evaluate serum bactericidal activity using bacterial ATP measurement as survival readout
Source: PLoS One. 2017 Feb 13;12(2):e0172163. doi: 10.1371/journal.pone.0172163 (PMC5305226; doi:10.1371/journal.pone.0172163)
Supplement: S1 Table — (PDF) [file pone.0172163.s001.pdf]

S1 Table. Raw values and calculated span in luminescence and CFU/mL for data shown in Fig 1A.

	CFU/mL	CPS	span in CFU/mL	span in CPS
T0	1.02E+03	0	1.98E+03	91
T180	3.00E+03	91		
T0	6.27E+03	59	1.25E+04	151
T180	1.88E+04	210		
T0	2.16E+04	296	1.06E+05	579
T180	1.28E+05	875		
T0	1.41E+05	1145	1.06E+06	4252
T180	1.20E+06	5397		
