# Supplementary figures and images for: Development of a high-throughput method to evaluate serum bactericidal activity using bacterial ATP measurement as survival readout
Source: PLoS One. 2017 Feb 13;12(2):e0172163. doi: 10.1371/journal.pone.0172163 (PMC5305226; doi:10.1371/journal.pone.0172163)

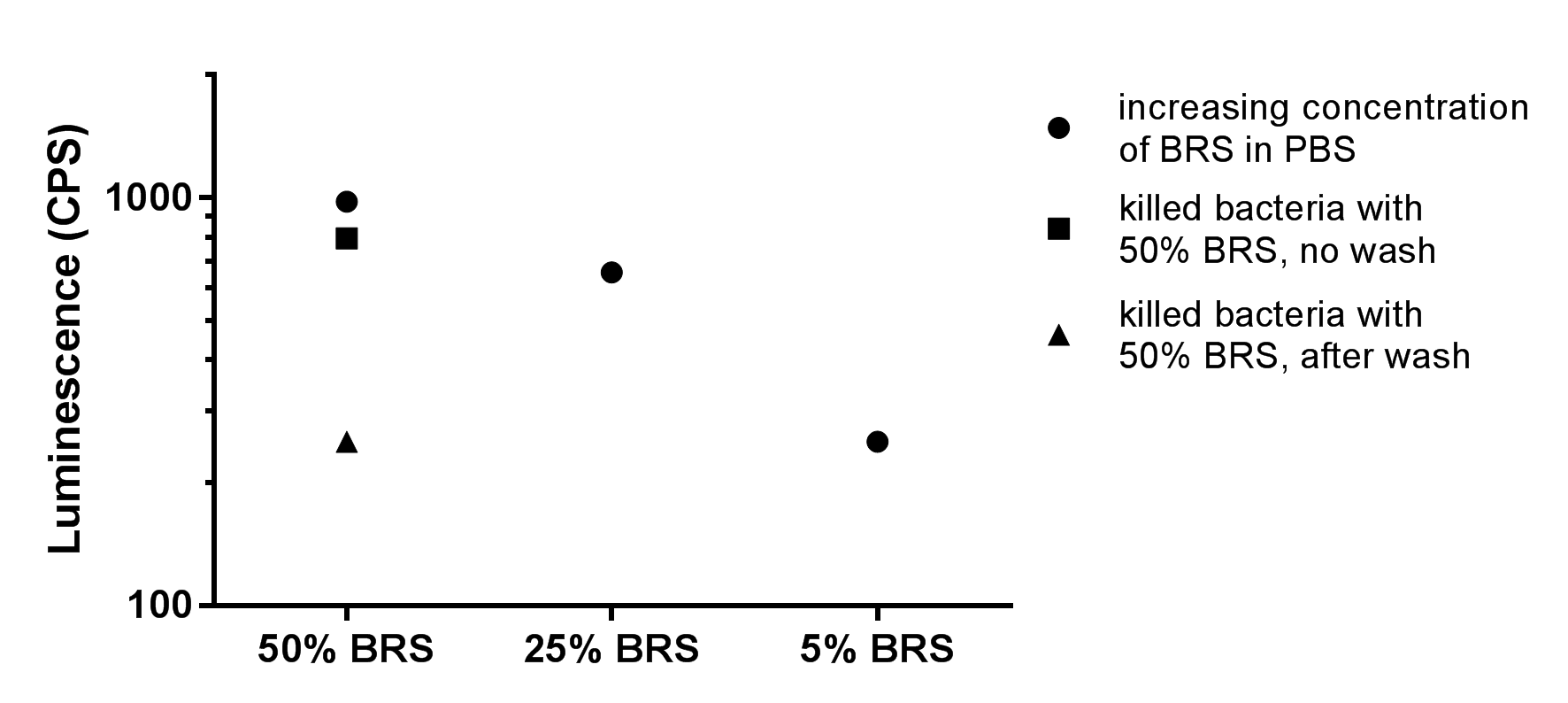

Supplement: S1 Fig — Without washing steps, increasing concentrations of BRS led to high luminescence values (up to 978 CPS using 50% BRS), compared to 5% BRS (252 CPS) (circle ●). Measuring by luminescence the killing effect of 50% BRS for C. freundii (inducing 99% killing), we observed that, without washing step, luminescence was overestimated (square ■), while one washing step decreased the value by almost three times (triangle ▲). (TIF) [file pone.0172163.s002.tif]
